# Endodontic Treatment of Two Calcified Mandibular Central Incisors: A Case Report

**DOI:** 10.7759/cureus.53066

**Published:** 2024-01-27

**Authors:** He Liu, Ahmed Hieawy, Ya Shen

**Affiliations:** 1 Division of Endodontics, Department of Oral Biological & Medical Sciences, University of British Columbia, Vancouver, CAN; 2 Division of Endodontics, Department of Oral Biological & Medical Sciences, Faculty of Dentistry, University of British Columbia, Vancouver, CAN

**Keywords:** dental operating microscope, cone-beam computed tomography, mandibular central incisor, calcified canal, endodontic treatment

## Abstract

Treating calcified root canals presents significant challenges, as incorrect approaches can result in treatment failure or lead to complications. The necessity for advanced diagnostic and therapeutic tools is often paramount in these situations. This case report demonstrates the successful treatment of two calcified mandibular central incisors, followed up for a period of up to six years. It emphasizes the effectiveness of integrating cone-beam computed tomography, dental operating microscopes, and ultrasonic instruments in the treatment of such challenging cases.

## Introduction

Pulp canal calcification is characterized by the deposition of mineralized tissue within the dental pulp [1,2]. It can manifest as small, distinct calcifications known as pulp stones or as extensive calcification, potentially leading to the narrowing or complete closure of the pulp canal, with its prevalence varying widely from 8% to 95% in different populations [1-4]. The exact cause of pulp canal calcification remains elusive, yet it is linked to a range of factors classified into physiological, pathological, and iatrogenic categories [2-4]. Physiological factors involve natural aging processes leading to secondary dentin deposition. Pathological stimuli, such as bacterial infections from caries or periodontal diseases, can trigger the body's defense mechanisms, resulting in calcification. Iatrogenic stimuli, associated with dental treatments like restorative procedures or pulp therapies, may inadvertently lead to calcification within the root canal [2-4]. These factors, contributing to pulp canal calcification, can significantly impact the anatomy and accessibility of the root canal system, thereby posing challenges to endodontic treatments [1,4,5].

Root canal treatment, the primary intervention for pulpal and periapical diseases, aims to eradicate bacterial infections, prevent reinfection, and promote healing of the affected periapical tissues [6-8]. Its success largely depends on the thorough cleaning and shaping of the root canals to remove diseased tissue, bacteria, and their byproducts [9-11]. This is followed by filling the canals with an inert material to prevent future microbial entry [12,13]. However, the presence of pulp canal calcification can complicate these procedures, challenging the effectiveness of the treatment by restricting access and complicating the cleaning and shaping process. This makes the management of calcified canals a critical aspect of successful root canal treatment [4,5].

Pulp canal calcification poses significant challenges in root canal treatment procedures, including the occlusion of the pulp chamber and obstruction of root canal orifices by dentin or calcified deposits [1,4,5]. This can result in missed canals, excessive dentin removal, reduced tooth fracture resistance, and potential perforations in the pulp chamber or pulpal floor [4]. Calcification can also narrow the root canal space, impeding thorough negotiation, shaping, cleaning, disinfection, and filling of the canal, increasing the risk of complications like ledges, canal perforations, or instrument separation. These complications highlight the need for advanced techniques and tools in the management of calcified canals [5].

This article discusses the endodontic treatment of two calcified mandibular central incisors with symptomatic apical periodontitis using ultrasonic instruments and a dental operating microscope. The use of these advanced tools facilitated the successful management of these challenging cases. The treated teeth remained asymptomatic and functional during a follow-up period of up to six years.

## Case presentation

A 28-year-old male patient was referred to our Department of Endodontics from a local dental clinic for the treatment of two calcified mandibular central incisors. He had been experiencing gingival and mucosal swelling for a week. The initial root canal treatment at the local clinic was unsuccessful in locating the canals in teeth #31 and #41 after creating the access cavity. The cavity was intentionally left open for drainage by the previous dentist who began the root canal treatment, and the patient was prescribed antibiotics, which successfully reduced the swelling. When he presented at our clinic one week later, his medical history revealed no significant issues. He was in overall good health, classified as American Society of Anesthesiologists (ASA) I, with no symptoms of systemic disease. Additionally, he maintained good oral hygiene and had no harmful or parafunctional habits.

**Figure 1:**
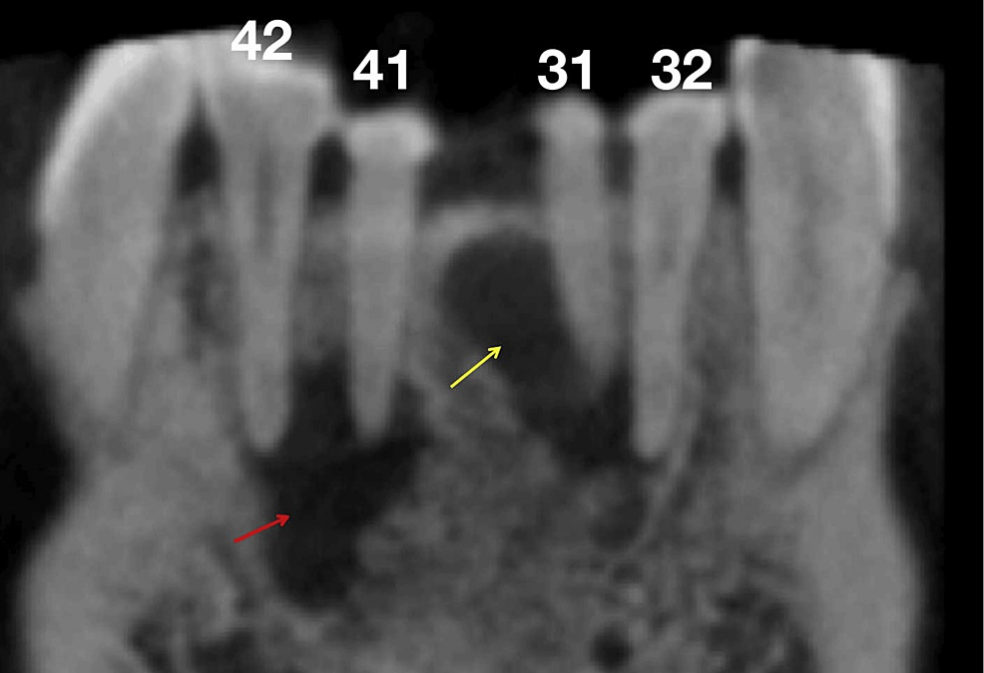
Preoperative coronal section of the cone-beam computed tomography (CBCT) image reveals large periapical lesions in teeth #31, #41, #32, and #42, indicated by red and yellow arrows

**Figure 2:**
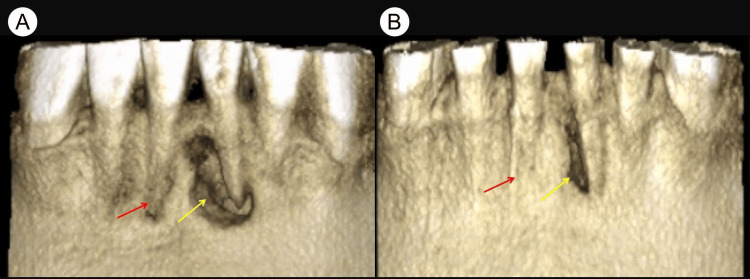
Preoperative and nine-month follow-up 3D reconstructions of cone-beam computed tomography (CBCT) images (A) The preoperative 3D reconstruction of the CBCT image reveals buccal bone plate fenestration in the apical areas of teeth #31 (yellow arrow) and #41 (red arrow). (B) The 3D reconstruction of the CBCT image from the nine-month follow-up shows significant healing of the periapical lesions in teeth #31 (yellow arrow) and #41 (red arrow).

**Figure 3:**
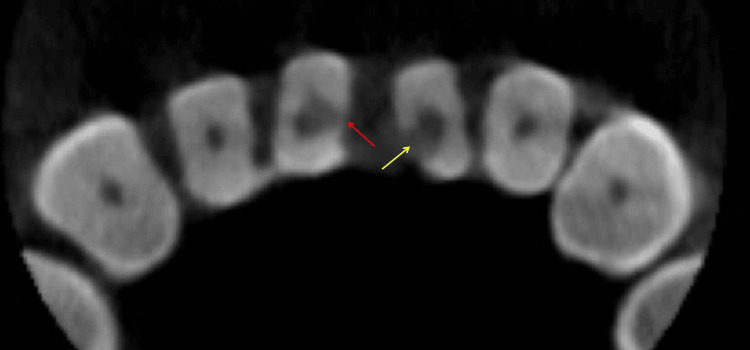
The preoperative cross-sectional cone-beam computed tomography image at the cementoenamel junction level reveals that the mesial aspects of the pulp chamber walls of teeth #31 (yellow arrow) and #41 (red arrow) were almost perforated

**Figure 4:**
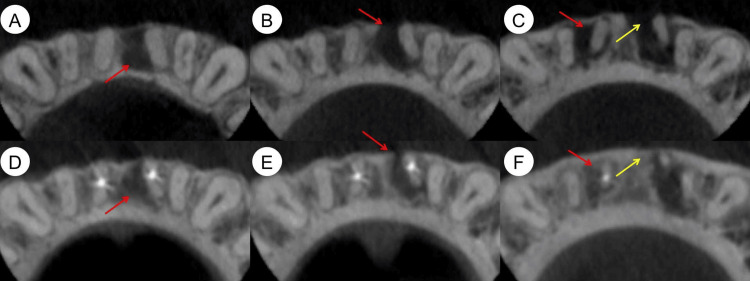
Preoperative and nine-month follow-up cross-sectional cone-beam computed tomography (CBCT) images (A-C) Preoperative cross-sectional CBCT images of the coronal (A), middle (B), and apical (C) thirds of tooth roots, showing periapical lesions (red arrows) and buccal bone plate fenestration (yellow arrow). (D-F) Nine-month follow-up cross-sectional CBCT images in the coronal (D), middle (E), and apical (F) thirds of the tooth roots, demonstrating significant healing of the periapical lesions (red arrows) and buccal bone plate fenestration (yellow arrow).

**Figure 5:**
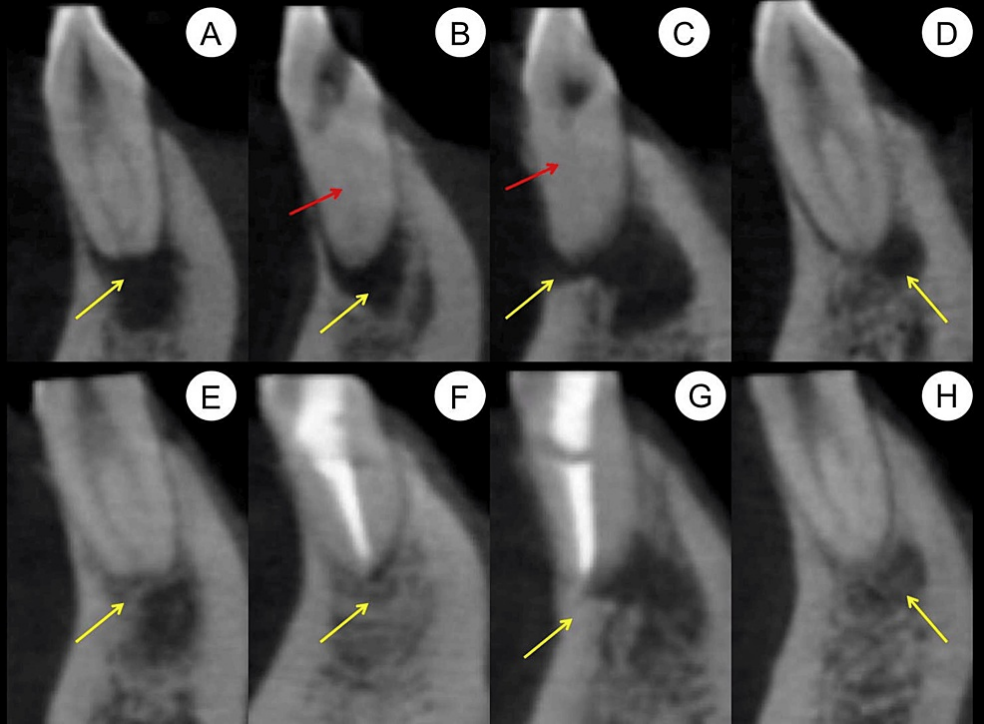
Preoperative and nine-month follow-up sagittal sections of cone-beam computed tomography (CBCT) images (A-D) The preoperative sagittal sections of the CBCT images display periapical lesions (yellow arrows) in teeth #42 (A), #41 (B), #31 (C), and #32 (D). The images also show that the root canals (red arrows) in teeth #41 (B) and #31 (C) were calcified and not visible. (E-H) The sagittal sections of the CBCT images taken at the nine-month follow-up demonstrate significant healing of the periapical lesions (yellow arrows) in teeth #42 (E), #41 (F), #31 (G), and #32 (H).

The clinical examination of teeth #31 and #41 revealed the absence of a temporary filling in the access cavity, normal tooth mobility, and a negative percussion test. Teeth #32 and #42 exhibited intact tooth crowns, normal mobility, and a negative percussion test. Slight swelling was noted in the mucosa around the apical area of teeth #31, #41, #32, and #42, but no sinus tract was present. Periodontal probing results were within normal limits. Cold testing with Endo-Frost cold spray (Roeko, Coltène/Whaledent Inc., Langenau, Germany) on teeth #31, #41, #32, #42, and control teeth (#11, #21, #12, #22) showed that the control teeth, along with #32 and #42, responded typically, indicating healthy pulp vitality. However, teeth #31 and #41 did not respond to the cold stimulus, suggesting pulp necrosis. Cone-beam computed tomography (CBCT) images revealed large periapical lesions associated with teeth #31, #41, #32, and #42 (Figure 1). The 3D reconstruction of the CBCT images showed buccal bone plate fenestration in teeth #31 and #41 (Figure 2A). The cross-sectional CBCT images at the cementoenamel junction level indicated near perforations in the mesial aspects of the pulp chamber walls of teeth #31 and #41 (Figure 3). Further, CBCT images in the coronal (Figure 4A), middle (Figure 4B), and apical (Figure 4C) thirds of these tooth roots showed extensive periapical lesions. The sagittal sections of the CBCT images highlighted the periapical lesions of teeth #42 (Figure 5A), #41 (Figure 5B), #31 (Figure 5C), and #32 (Figure 5D). The root canals of teeth #31 and #41 were severely calcified and not visible in the imaging. Based on these findings, teeth #31 and #41 were diagnosed with previously initiated and acute apical abscesses. The proposed treatment plan included root canal treatment followed by composite resin restoration. The patient was fully informed about the treatment plan and procedures and gave his consent.

**Figure 6:**
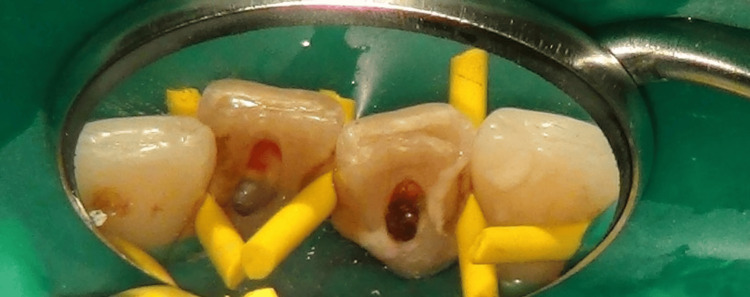
Microscopic photo taken when the rubber dam isolation was completed

**Figure 7:**
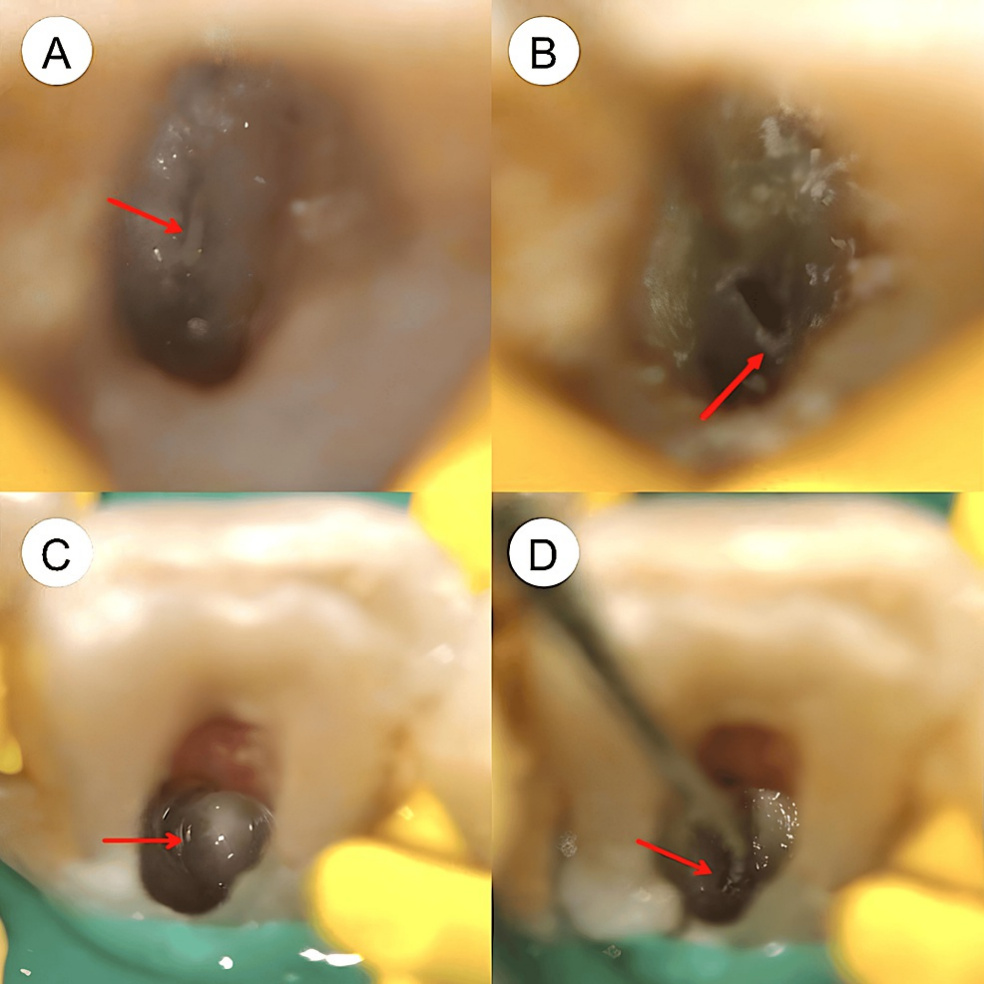
Microscopic photos taken during the root canal treatment procedures (A) There was calcification (red arrow) on the pulp chamber floor of tooth #31. (B)The root canal orifice (red arrow) in tooth #31 was successfully located. (C) There was calcification (red arrow) on the pulp chamber floor of tooth #41. (D) The root canal orifice (red arrow) in tooth #41 was successfully located.

After isolating teeth #31 and #41 with a Hygenic Dental Dam (Coltène Whaledent, Altstätten, Switzerland), the pulp chambers of these teeth were examined using a DG16 endodontic probe (Hu-Friedy, Chicago, USA) under a dental operating microscope (DOM) (OPMI PICO; Carl Zeiss, Oberkochen, Germany) (Figure 6). The examination revealed white calcifications on the pulp chamber floors of teeth #31 and #41. To remove these calcifications, ultrasonic tips, specifically ET21 and ET25 (Satelec Acteon, Merignac, France), were employed. For canal negotiation, C-Pilot files (VDW, Munich, Germany) were used (Figure 7). The root canal working lengths were determined using an electronic apex locator (J Morita Corp, Tokyo, Japan), and verified through measurements on the sagittal section of the CBCT images. The shaping of the canals was carried out using the Twisted Files NiTi rotary system (TF; SybronEndo, Orange, USA). This process included alternating irrigation with a 3% sodium hypochlorite (NaOCl) solution and a 17% ethylenediaminetetraacetic acid (EDTA) solution. The canals were then soaked in the 3% NaOCl solution, followed by three 20-second irrigation sessions using an Irri-Safe ultrasonic tip (Satelec Acteon). Subsequently, they were irrigated with a 17% EDTA solution, employing the Irri-Safe ultrasonic tip for three additional 20-second sessions. After these treatments, the canals were thoroughly rinsed with sterile water and dried using paper points. Calcium hydroxide paste (Pulpdent™ paste; Pulpdent Corporation, Watertown, USA) was then introduced into the canals. The procedure concluded with the access cavity being securely sealed with a temporary filling material.

**Figure 8:**
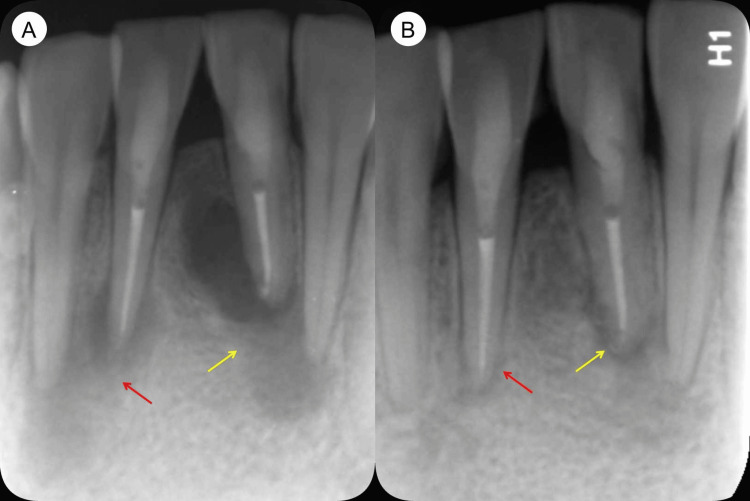
Post-obturation and 28-month follow-up periapical radiographs (A) The post-obturation periapical radiograph reveals the presence of large periapical lesions in teeth #31 (yellow arrow) and #41 (red arrow). (B) A periapical radiograph taken at the 28-month follow-up shows that the periapical lesions in teeth #31 (yellow arrow)and #41 (red arrow) have significantly healed.

The patient returned for a follow-up visit one week later and reported that teeth #31 and #41 were asymptomatic. During this visit, the temporary filling was removed, and the canals were irrigated with a 3% NaOCl solution. The calcium hydroxide paste was then removed using the Irri-Safe ultrasonic tip (Satelec Acteon), followed by a trial fitting of the main gutta-percha cones. The fit of the heat carrier tip, Buchanan hand pluggers, and the needle of a 23G gutta-percha capsule, all from SybronEndo (Orange, USA), was carefully checked. The canals were irrigated using the same protocol as in the initial visit. After the irrigation process, paper points were used to dry the canals thoroughly, ensuring the removal of any residual moisture or leftover irrigation solution. Before placing them in the canal, the tip of the gutta-percha cones was dipped in a small amount of AH Plus sealer (Dentsply, Maillefer, Germany). The root canal filling was completed using the continuous wave obturation technique with the Elements Obturation Unit (SybronEndo). A periapical radiograph was then taken to evaluate the quality of the root canal filling (Figure 8A). Finally, the tooth restoration was completed using Filtek Z350 light-cured composite resin (3M ESPE, St. Paul, USA).

At the nine-month follow-up examination, the patient's teeth #31 and #41 continued to be asymptomatic. The 3D reconstruction of the CBCT image revealed that the periapical lesions had significantly resolved (Figure 2B). Cross-sectional CBCT images in the coronal (Figure 4D), middle (Figure 4E), and apical thirds (Figure 4F) of the tooth roots showed that the large periapical lesions along the roots of teeth #31, #41, #32, and #42 had also significantly resolved. The sagittal sections of the CBCT images indicated complete healing of the periapical lesions in teeth #32 (Figure 5H) and #42 (Figure 5E) while those in teeth #31 (Figure 5G) and #41 (Figure 5F) had significantly resolved. At the 28-month follow-up, teeth #31 and #41 remained asymptomatic. A periapical radiograph taken at this time showed significant healing of the periapical lesions in these teeth. Furthermore, during a follow-up phone call six years after the initial treatment, the patient reported that teeth #31 and #41 continued to be asymptomatic and functional (Figure 8B).

## Discussion

The preoperative diagnosis of canal calcification is essential for the successful and efficient execution of root canal treatment, as it assists in devising an appropriate strategy and selecting the right instruments to handle calcified canals [4,5]. Understanding the degree of calcification is also key in evaluating the complexity of the procedure and the potential for complications [5]. Periapical radiographs, being two-dimensional representations of three-dimensional structures, often have significant overlap and provide limited data [14]. In contrast, CBCT is a three-dimensional imaging technique that significantly enhances endodontic diagnosis and treatment planning [14]. Limited field-of-view CBCT specifically can pinpoint the location and severity of calcification with greater precision [14]. In our case, CBCT sagittal sections showed that teeth #31 and #41 were almost entirely calcified, with no discernible canals in the roots. Additionally, limited field-of-view CBCT analysis can be instrumental in three-dimensionally measuring the distance from the obstructed orifice to reference points and determining the thickness of the blockage [5]. This method enables more accurate procedures, reducing the risk of damaging tooth structure and avoiding perforation of the pulp chamber floor or the root canal [5,14]. Nevertheless, it is crucial to adhere to the "as low as reasonably achievable (ALARA)" principle to prioritize patient safety [14].

The successful location and treatment of all root canals are crucial for the effectiveness of root canal treatment, as missing any canal is significantly linked to the occurrence of post-treatment apical periodontitis [15]. The DOM is essential in this regard, offering high magnification and illumination levels [16-19]. This enhanced visibility is particularly critical for obtaining a clear and detailed view of the pulp chamber floor, which is instrumental in identifying subtle canal openings, especially in teeth with calcified or complex canal systems [16-19]. In cases of pulp canal calcification, the DOM proves invaluable for locating canals that are calcified or otherwise obscured [17]. Compared to the naked eye and surgical loupes, the DOM has shown significantly greater effectiveness in detecting orifices [18]. Furthermore, the DOM also facilitates the negotiation of root canals. For instance, when working without magnification, mesiobuccal second (MB2) canals in maxillary molars were located in 93% (42 teeth) and negotiated in 69% (31 teeth). However, with the use of the DOM, the MB2 canal was located in an additional tooth (increasing the total to 96%) and negotiated in five more teeth (raising the total to 80%) [19]. This underscores the significant advantage of using the DOM in enhancing the efficacy of root canal location and negotiation. 

In root canal treatment, differentiating between reparative dentin or calcifications and the pulp chamber floor is essential, as the former are often lighter and can obscure the latter and its orifices [20]. Utilizing a DOM is vital for this purpose. In our case, under the DOM, tooth #31 exhibited light calcification at the center, surrounded by a darker line of the pulp chamber floor, creating a 'bullseye' appearance (Figure 7A). The calcifications were efficiently removed using an ultrasonic tip, enabling the root canal to be located and negotiated easily with the C-Pilot file. In tooth #41, the pulp chamber floor appeared calcified and shiny under the DOM (Figure 7C). After removing the calcification from the pulp chamber floor, the canal was successfully located and negotiated.

In recent years, the use of DOM and ultrasonic instruments in root canal treatment has markedly improved the success rate in managing calcified root canals [17,21]. Wu et al. assessed the effectiveness of combining DOM and ultrasonic instruments in handling complex root canal treatment cases [17]. In their study, 345 teeth with a total of 546 root canals, which were unmanageable by conventional methods, underwent treatment using DOM and ultrasonic instruments by the same team of endodontists. The success rate for calcified canals was 74.0%.

Recently, guided endodontics has been proposed for treating calcified canals, especially for the anterior teeth [22]. This approach enhanced precision due to the use of 3D imaging and printed guides, which significantly reduced the risk of perforation and structural damage. This approach also improves efficiency and predictability, streamlining the treatment process [23,24]. However, there are notable drawbacks such as the high cost and limited accessibility of the necessary technology. Additionally, there's a learning curve for dentists to effectively utilize these advanced techniques [23,24]. Despite these challenges, guided endodontics represents a significant advancement in managing calcified root canals, although its application should be carefully weighed against its limitations and the specific context of the clinical environment.

## Conclusions

In this case report, the successful treatment of two calcified mandibular central incisors, with a follow-up period extending up to six years, exemplifies the effectiveness of integrating CBCT, DOM, and ultrasonic instruments in managing such challenging cases.
